# Specifying Inputs for the Computational Structure of a Surgical System via Optical Method and DLT Algorithm Based on In Vitro Experiments on Cardiovascular Tissue in Minimally Invasive and Robotic Surgery

**DOI:** 10.3390/s22062335

**Published:** 2022-03-17

**Authors:** Grzegorz Ilewicz, Edyta Ładyżyńska-Kozdraś

**Affiliations:** Faculty of Mechatronics, Warsaw University of Technology, ul. św. Boboli 8, 02-525 Warsaw, Poland; edyta.ladyzynska@pw.edu.pl

**Keywords:** surgical system, RCM structure, endoscopy, in vitro, cardiovascular tissue, transient states, jerk-movement, CMOS sensor, APAS, DLT algorithm, PID, DC Motor

## Abstract

With the application of four optical CMOS sensors, it was possible to capture the trajectory of an endoscopic tool during an in vitro surgical experiment on a cardiovascular preparation. This was due to the possibility of obtaining a path when a reflective marker was attached. In the work, APAS (Ariel Performance Analysis System) software and DLT (direct linear transformation) algorithm were used. This made it possible to acquire kinematic inputs to the computational model of dynamics, which enabled, regardless of the type of surgical robot structure, derivation of the analogous motion of an endoscopic effector due to the mathematical transformation of the trajectory to joints coordinates. Experiments were carried out with the participation of a practiced cardiac surgeon employing classic endoscopic instruments and robot surgical systems. The results indicated by the experiment showed that the inverse task of kinematics of position for the surgical robot with RCM (remote center of motion) structure was solved. The achieved results from the experiment were used as inputs for deriving a numerical dynamics model of surgical robot during transient states that was obtained by applying the finite element method and by driving dynamics moments acquired through the block diagrams method using a steering system with DC (direct current) motor and PID (proportional–integral–derivative) controller. The results section illustrates the course of kinematic values of endoscopic tools which were employed to apply numerical models as inputs, the course of the driving torque of the model of the surgical robot that enabled the selection of the drive system and the strength values, stresses and displacements according to von Mises hypothesis in its structure during the analysis of transient states that made it possible to establish the strength safety of the surgical robot. For the conducted experiments, the accuracy was ±2 [mm]. In the paper, the employment of optical CMOS sensors in surgical robotics and endoscopy is discussed. The paper concludes that the usage of optical sensors for determining inputs for numerical models of dynamics of surgical robots provides the basis for setting the course of physical quantities that appear in their real object structure, in manners close to reality.

## 1. Motivation

Deformable and non-deformable (built of rigid bodies) numerical models of surgical robots are used for inexperienced determination of the physical quantities appearing in their real object mechanical structure. The stresses affecting the safety factor, displacements related to the accuracy of the effector positioning, the natural frequencies which specify the size of the vibration amplitude during the resonance phenomenon and many more attributes can be considered as very vital factors in much scientific research. The mentioned physical quantities allow for scientific inference about physical phenomena occurring in the structure of a surgical robot via a numerical method. The results from computational models are cheaper to produce than those obtained through experimental research due to the fact that no expensive measuring equipment is needed. Many physical quantities can only be determined numerically (e.g., for new honeycomb structures, composite materials, sandwich materials which have not been produced so far, for materials with any fracture, for certain systems of applied forces). Experiments may be difficult to perform, for example, due to long waiting periods for the results. It is also recognized that a correctly created numerical model makes it possible to avoid typical errors during experimental measurements (e.g., human error) and, at the same time, gives the correct quantitative and qualitative results.

A significant quantity in every computational numerical model is the input, i.e., the signal fed into its input, which can also be named as the setpoint or control quantity. It is difficult to analytically determine the magnitude and the course of input to the numerical model because it should correspond to the actual forces, velocities, accelerations, and jerk-like motions in which real bodies (gears, motors) act on the mechanical systems of a surgical robot. A particularly important input, from the benefits point of view in a surgical robot, is the velocity input, since the surgeon, who conducts the operation from the point of view of this physical quantity, performs positioning and operational activities with endoscopic tools relative to the tissue. The surgical robot should thus move at velocities assumed by the surgeon. It is recognized then that the appropriate functionality of the robot is ensured. This means that it moves according to the surgeon’s assumptions. Herein, the activities of the surgical robot can be divided into two groups: related to the positioning of the endoscope in relation to the tissue and related to the interaction of the surgical robot and the tissue and its incision.

The aim of this article is to determine the input velocity signals for two numerical models of a surgical robot based on in vitro surgical experiments on cardiovascular tissue. In order to increase the similarity of the numerical models to the real object, which is a surgical robot, DC motor models and control systems based on PID regulators were added to the mechanical structure. In this method, the aim is to obtain the course of the output quantities that are close to reality, such as driving moments and stresses and deformations during the analysis of transient states. The results obtained from numerical models are of significant importance in the design and construction process before building a real object. The article fills an important gap in research, which is the lack of determination and application of kinematic parameters from in vitro experiments on the cardiovascular tissue with the usage of endoscopic tools and optical CMOS (complementary metal-oxide-semiconductor) sensors for numerical dynamics models in order to obtain close to reality characteristics of physical quantities that appear in the structures of surgical robots. Testing numerical models with the use of input velocities obtained in the in vitro experiment allows establishing whether they are within the strength-safe ranges.

The safety of the robot from the strength point of view can be characterized by the value of the safety factor, which in this work is defined as the yield point of the adopted construction material to the ratio of the maximal reduced stress according to the von Mises hypothesis. There is a clear mechanical relationship between velocity and safety of the robot. Due to the velocity changes in individual joints, additional inertial loads appear in the robot structure that affect the value of the safety factor. The presented numerical models enable inferring the strength and the driving torque courses of individual degrees of freedom. The obtained results allow for the selection of a drive system in the form of a DC motor in the case of a numerical model of driving moments, and determination of strength values significant from the point of view of the strength safety of a surgical robot in the case of a transient model formulated on the basis of the finite element method.

In the study, a series of experiments were carried out using optical sensors and the APAS software to analyze two-dimensional signals from the four CMOS (complementary metal-oxide-semiconductor) sensor matrix. Here, information on surgical procedures on animal tissue, i.e., cardiovascular tissue (*Sus scrofa. F domestica*), performed by an experienced surgeon using endoscopic instruments was incorporated. The courses of physical quantities concerning the robot links were also recorded, enabling their use as input for numerical models. Moreover, to implement the data from optical experiments into numerical models, the inverse task of kinematics in a surgical robot had to be solved with a remote center of motion mechanism. The article additionally contains examples of the usage of optical sensors in surgical robots regarding various structures applied in endoscopic operations.

The article focuses on the description of the use of CMOS optical sensors to obtain the kinematic values of endoscopic tools in the field of medicine that is extremely difficult from the point of view of the specificity of treatments, i.e., minimally invasive cardiac surgery. During the in vitro experiment on cardiovascular tissue, the ultramodern structure of the medical robot “Robin Heart Vision” was used, therefore, an extended minimally invasive experiment is shown compared to the traditional—where the endoscopic camera is controlled by an assistant cardiac surgeon. The paper provides an answer to the question of how to obtain the kinematic parameters that will serve as inputs for the numerical models that need to be solved in the process preceding the experimental validation of a prototype of a surgical robot for heart surgery in the MIRS technique (minimally invasive robotic surgery) using optical sensors. The modern optical sensors Basler A602fc-2 recording the image of the operating field at a frequency of 100 [Hz] and the commercial APAS software applied in biomechanics were used to detect the reflective marker. By way of utilizing these sensors, we found that during the movement of endoscopic instruments, jerk-like motions appear, which may lead to the negative phenomenon of tearing the tissue. The obtained results will contribute to the construction of a medical robot that moves in a smooth manner, where there are no negative jerk-like motions affecting the tissue that is operated upon.

## 2. Literature Review

Up to the present moment, researchers have employed three main methods for determining the kinematic quantities that are applicable as inputs to the models of dynamics in a surgical robot, i.e., that based on optical sensors, accelerometers, and three axis gyroscopes and OTS (optic test station) systems. For example, in work [1], operations with the use of surgical robot and classical instruments were analyzed, and parameters such as the length of treatment, tool path length, and tool orientation were assumed to be important from the operation analysis point of view. Two optical sensors were used to determine the parameters related to the movement. In work [2], an optical system was employed to analyze the redundancy in the structures of surgical robots during orientation and positioning tasks. An experiment with the use of optical sensors for the analysis of a surgical operation using an innovative method, without the use of reflective markers, was carried out in work [3], where the courses of velocity and acceleration of the surgeon were determined to objectively assess the surgical technique. Furthermore, a 3D optical motion-analyzing system has been applied to determine the trajectory of robotic endoscopic tools in [4]. Accordingly, passive markers were attached to a laparoscopic instrument. Three CCD (charge-coupled device) matrices were used in these experiments. The EVA (endoscopic video analysis) system for examining the kinematics of endoscopic instruments with the optical method is shown in work [5]. Therein, spatial charts of tool paths were assessed.

In the described works, only one or two optical sensors were used to determine kinematic parameters. In contrast, in the innovative experiment analyzed in this study, four optical sensors with a high sampling frequency of 100 Hz were employed. This resulted in greater accuracy with regard to the obtained outcomes in the assessment of endoscopic tool trajectories.

The works [6,7,8] describe the photogrammetric method with the application of the APAS system, and take into account the accuracy of the obtained results. A comprehensive description of experiments with the use of optical sensors carried out at the Professor Zbigniew Religa Foundation of Cardiac Surgery Development Laboratory of Biocybernetics on Robin Heart robots during minimally invasive experiments is shown in work [9]. This work also includes a comprehensive and interesting study about surgical robotics.

In most of the analyzed studies, the researchers limited themselves to determining velocity as being an important parameter for the surgeon to objectively evaluate the technique of performed surgical procedures. In this study, acceleration is also considered an important parameter for the assessment of surgical skill, as it determines the inertia of biomechanical systems such as the surgeon’s hand—endoscopic tool—trocar—operated tissue. The second important kinematic parameter analyzed in this paper, wherein its definition is new in relation to the knowledge available today, is jerk-movement, defined as a physical quantity that is the third derivative of the trajectory. It is a quick, sharp sudden movement that prevents the smooth operation of endoscopic tools and robot mechanisms.

An alternative method of determining the trajectory based on integration of the acceleration signal is shown in works [10,11]. Accelerometers and IMU (inertial measurement unit) with three axis gyro sensors were used in these works to determine the acceleration. OTS systems were also applied to ascertain the trajectory during various operations [12,13,14]. The disadvantages of IMU systems include low accuracy and drift error (DC bias). The drawbacks of the OTS system include limited measuring volume and occlusion, as well as low measurement frequency. In addition, the disadvantage of using the described sensors to objectively evaluate the technique of the performed surgical procedure is the lack of simultaneous observation of the performed procedure on the monitor, along with the marked trajectory on which the minimally invasive or endoscopic operating tool of the surgical robot moves. This makes it difficult to assess the correctness of the surgical technique applied. These disadvantages do not exist in the proposed method.

The paper [15] illustrates a method of solving the inverse kinematics problem about the position using the PSO algorithm (particle swarm optimization) for the creation of a surgical robot with three linear and three rotary axes that is intended to provide treatment of fractures. The iterative solution of inverse kinematics of a da Vinci robot in a controller-responder system using the Newton method is shown in work [16], while the inverse kinematics solution of a surgical robot with six degrees of freedom implemented upon a model in CATIA program and D-H (Denavit and Hartenberg) notation is shown in work [17]. Beyond the aforementioned, a comprehensive analysis of dynamics based on the energy method of a robot with a remote center of motion mechanism is shown in work [18].

In comparison with the above works, this work extends the dynamics models with DC motors and control systems built upon PID controllers. The numerical models considered in the study are therefore closer to reality from the point of view of taking into account all the structural elements of the real surgical robot.

The work [19] illustrates the application of the finite element method to the da Vinci surgical robot, which resulted in obtaining physical quantities related to resonance. The basis of the frequency analysis is static analysis. Its results, however, have not been shown in the article. The finite element method for testing the strength properties of a surgical robot tool is illustrated in [20]. Moreover, the values of stresses and deformations calculated based on the von Mises hypothesis using the finite element method of a laparoscopic tool with four degrees of freedom are illustrated in [21]. With the employment of numerical methods in the described works, results were obtained which made it possible to obtain a structure for surgical operations that is safe from the point of view of strength.

In this work, through the usage of sensors, the courses of trajectories, velocities, accelerations, and jerk-like movements occurring during minimally invasive operations were generated. The obtained waveforms were then applied as input quantities to the numerical models used in the work. The outcome of this work are the results in the form of torque waveforms of a numerical model made in the Simulink program and the strength values of the mesh model generated by FEM (finite element method) during transient analysis. These form the basis for obtaining a safe mechatronical object—a surgical robot.

The article deals with the scientific problem of measuring the kinematic values of surgical robots and minimally invasive tools using a system of modern CMOS optical sensors correlated to measure the trajectory by means of applying the APAS motion analysis system. Similar experiments, as shown in the literature review, are of interest to researchers of biomedical engineering and cardiosurgery. In this article, we demonstrate that the use of optical sensors for devices related to minimally invasive medicine is an attractive solution enabling the acquisition of kinematic parameters as inputs for computational numerical models. It should be underlined that optical systems are of great use in research related to surgical robots from the point of view of discerning displacements that appear in their structures during their operation in the operating field. These displacements are relatively large. At the same time, due to the sampling frequency of the 100 Hz CMOS sensors used, they make it possible to study the phenomenon of mechanical vibration, which is very important for prototype surgical robots. The research area of this paper that is discussed above broadens the field of research by way of applying CMOS optical sensors (devices that differ in the methodology of application) rather than IMU accelerometric sensors or OTS systems in research related to minimally invasive cardiac surgery.

## 3. Experiments with the Use of Optical CMOS Sensors in Surgical Robotics and Minimally Invasive Surgery

**Figure 1:**
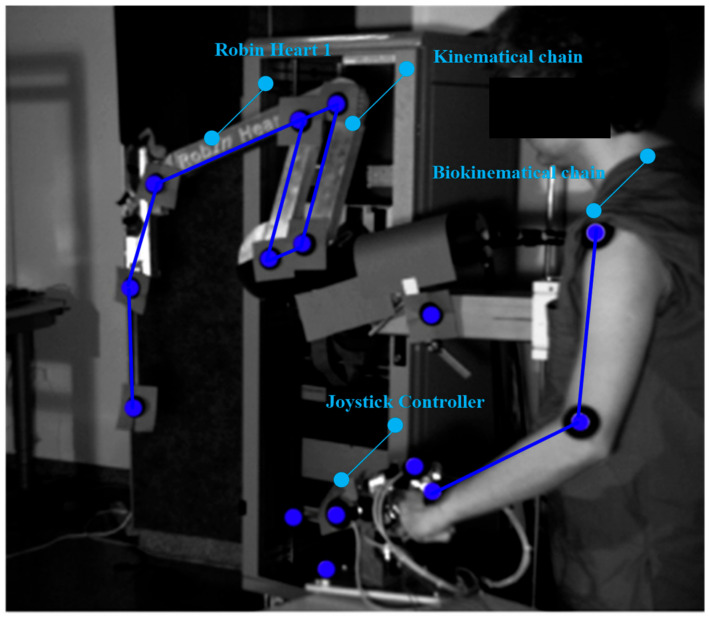
Robin Heart 1 surgical robot for soft tissue surgery, endoscopic controller and robot operator with reflective markers attached to the biokinematical chain.

In the experimental sphere, in vitro tests with the use of optical CMOS sensors were carried out. These concern surgical robots equipped with tools for operating upon soft tissue. The research was undertaken in the Professor Zbigniew Religa Foundation of Cardiac Surgery Development Laboratory of Biocybernetics to obtain information on the kinematic quantities appearing in the structure of a surgical robot. A robot (teleoperator) useful for operating upon soft tissue in the laboratory and in the operating room, Robin Heart 1 is shown in Figure 1 [22,23].

In our study, reflective markers were attached to the joints of the robot’s remote center of motion mechanism (RCM mechanism) to obtain joint movement and the trajectory of the endoscopic tool. After registering the movement, it was possible to obtain the spherical kinematics in the surgical robot using APAS software and the DLT algorithm. Reflective markers were also attached to the endoscopic controller and the operator’s biokinematic chain to acquire the trajectories of the upper limbs. The movements of the robot and the operator were recorded using two optical CMOS Basler A602fc-2 devices, at a frequency of 100 Hz. The conducted experiments were intended to check the gain value between the motion generator system and the surgical robot system in the controller-responder approach. In this method, it became easy to say whether the assumed re-scaling displacement of the surgeon’s hand corresponds to the actual displacement of the operating instrument. Therefore, it was possible to test the safety of the surgical robot for given scale factors.

The reflective markers placed on the joints of the operator’s upper limb are illustrated in the Figure 1. Based on the trajectory of the markers, conclusions were drawn about the ergonomics of the surgeon during steering, as well as the usage of the endoscopic controller.

**Figure 2:**
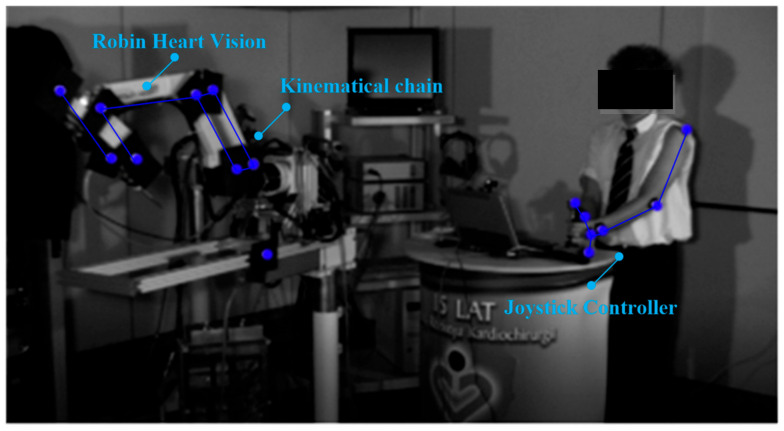
Robin Heart Vision surgical robot for soft tissue surgery and joystick controller and robot operator with reflective markers attached to the biokinematic chain.

Experiments with CMOS sensor systems were also carried out on the Robin Heart Vision robot equipped with an endoscope, whereby the surgeon can view the operated organ after the endoscope has been inserted through the access port into the space around the operated organ. Such a robot is controlled by a joystick motion controller, as shown in Figure 2.

**Figure 3:**
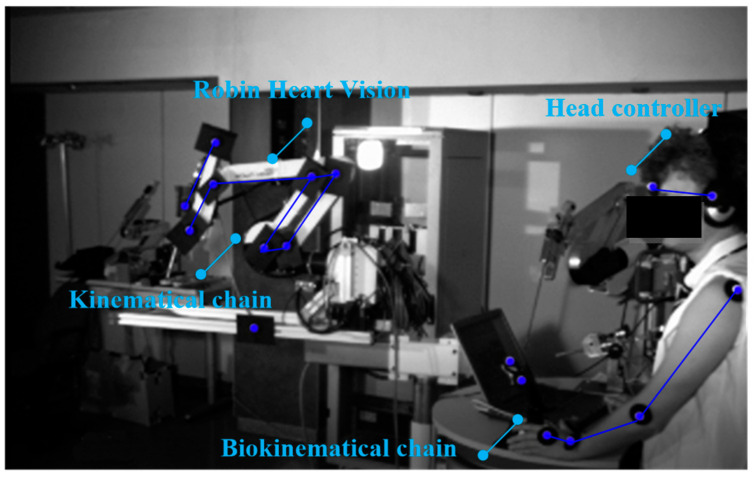
Robin Heart Vision surgical robot for soft tissue surgery and head controller and robot operator with reflective markers attached to the biokinematic chain.

The robot is also controllable by a modern method based on an electronic system attached to the operator’s head. Here, the endoscope attached to the structure of the Robin Heart Vision surgical robot moves in accordance with the surgeon’s head movements, as shown in Figure 3.

The research carried out in the field of surgical robotics made it possible to experimentally confirm the suitability of the use of surgical robots for operations within the given parameters. The assumed laboratory functionality was obtained for the assessed displacement values. The constant point kinematic nature of the robot systems equipped with a remote center of motion mechanism was confirmed. The correct operation of the controller-responder systems for the set displacement values was also confirmed. Experimentally obtained facts using CMOS sensors and APAS system make it possible to conclude that numerical models of the dynamics of surgical robots based on a remote center of motion will be useful in numerical analyses of physical quantities appearing during tissue surgery.

## 4. Materials and Methods

**Figure 4:**
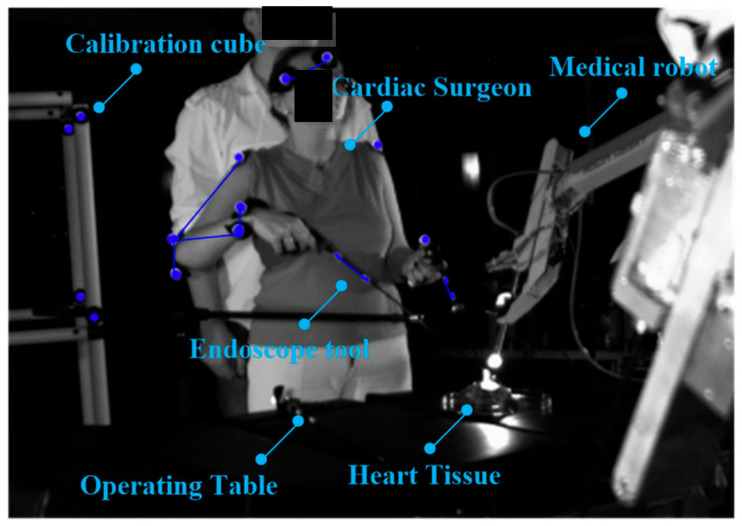
Surgeon with two endoscopic instruments and the surgical robot “Robin Heart Vision” with an endoscopic camera. The surgeon is operating upon heart tissue (*Sus scrofa f. domestica*) and observes the operations on the monitor above the robot.

**Figure 5:**
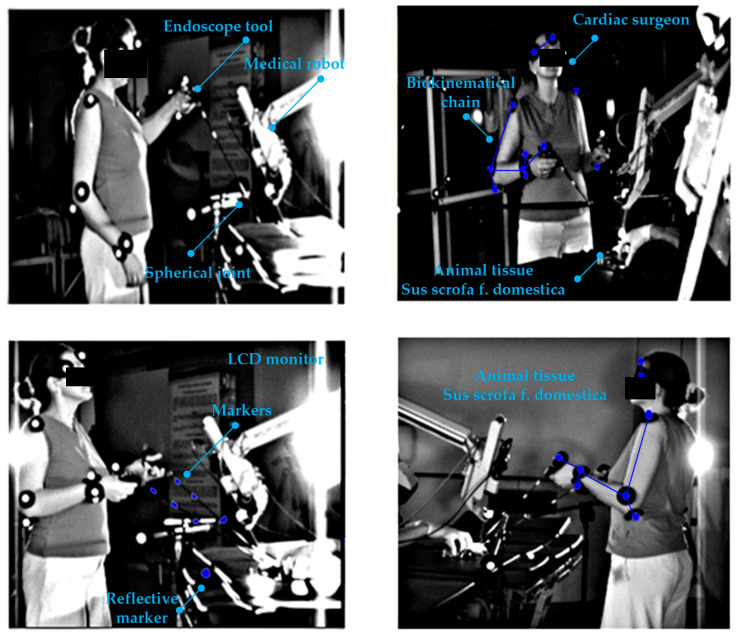
Endoscopic in vitro experiment on an animal preparation with the use of the surgical robot “Robin Heart Vision” and endoscopic instruments. Four optical CMOS sensors are used to acquire information about endoscopic tools displacements and the biokinematical chain of the cardiac surgeon.

A surgeon operating upon a cardiovascular tissue (*Sus scrofa F. domestica*) preparation with endoscopic tools is illustrated in the Figure 4 and Figure 5. Herein, the surgeon observes the operated tissue on a monitor, which is viewed from the endoscopic camera of the Robin Heart Vision used for this purpose. The surgeon’s movement is recorded at 100 [Hz], using the four digital sensors of an CMOS Basler A602fc-2. The conditions of the in vitro experiment on cardiovascular tissue closely correspond to the true situation of minimally invasive heart surgery. The medical trocar was simulated by means of a spherical joint, actual endoscopic tools employed in performing operations were exploited, the clinically tested Robin Heart Vision medical robot was used, the cardiovascular tissue was on a professional operating table, the operation was performed by an experienced cardiac surgeon with many years of experience. In view of the above, one should be convinced that the obtained kinematic parameters based on the data from the optical sensor system correspond to those that appear during surgery on the pathologically changed tissue of the patient. This work may be of interest to designers of state-of-the-art devices such as medical robots.

**Figure 6:**
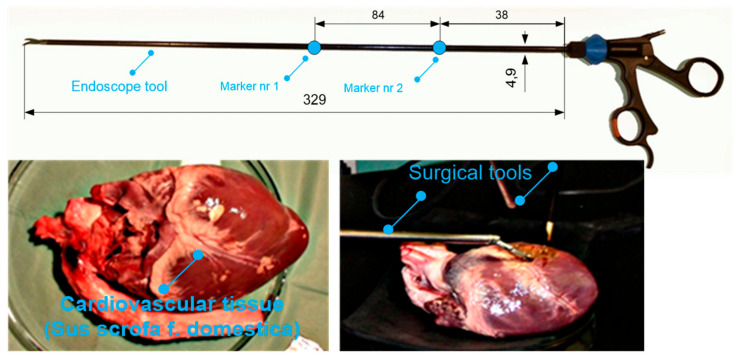
An endoscopic tool with four degrees of freedom when it is moved through the trocar and cardiovascular tissue on which the experiments were carried out with the use of an optical system.

**Figure 7:**
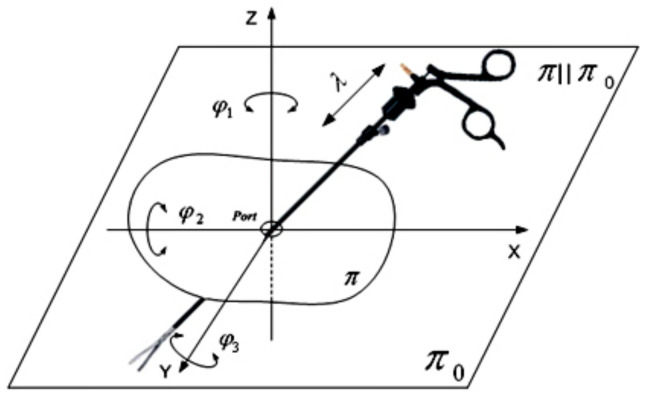
Endoscopic tool with the description of the degrees of freedom in space: three rotations and translations after passing through the port—an access opening in the outer shell of the body.

During the experiment, two endoscopic tools with two markers were used to perform set-up and positioning activities on the cardiovascular tissue shown in Figure 6. The endoscopic tools had four degrees of freedom after passing through the access port. The described configuration of the degrees of freedom can be read from Figure 7.

The method applied to determine the physical quantities of mechanical objects in motion is based on placement of reflective markers at vital points of the biomechatronic object (joints) and recordings of the movement with a set of at least two optical sensors, as well as the usage of DLT matrix transformations and the FDM (finite difference method) to calculate trajectories and time derivatives of trajectories. The recording of movement consists of registering light radiation mirrored from a reflective marker by the CMOS sensor matrix. In this method, at a given sampling frequency, the CMOS matrix determines the number of positions in one-second time intervals.

**Figure 8:**
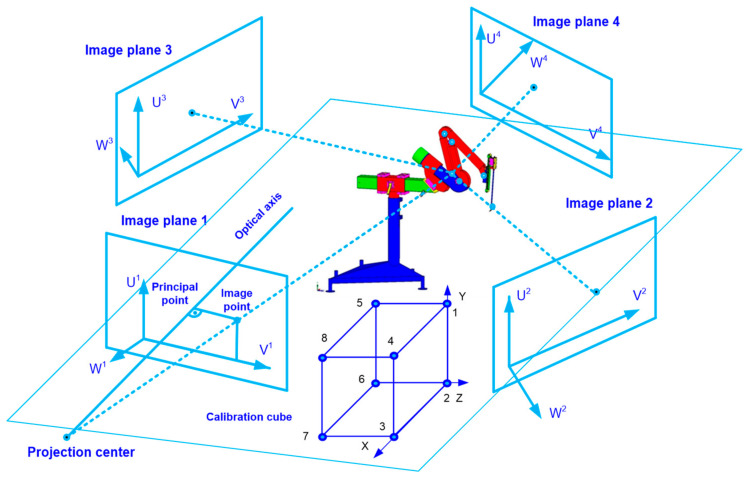
The layout of the experiment. It involves a system of four CMOS optical sensors, a calibration cube, and the surgical robot “Robin Heart 1” as the object of the experiment. The parameters of DLT algorithm are marked on the image plane of every CMOS sensor.

The measurement method illustrating the experiment using four optical sensors is shown in Figure 8. The calibration cube allows determining the coordinates based on which the APAS motion analysis system correlates data from the four optical sensors into the trajectory. Eight reflective markers at a distance of 1 [m] were placed on the cubic calibration cube.

For the assumed volume of 2 × 2 × 1.5 [m] of the experimental volume, an accuracy of 2 [mm] was obtained using the APAS system. This value was confirmed by measuring the distance between two reflective markers on the rigid sleeve of the endoscopic instrument by applying a linear measure. The interpretation of the above statement is as follows. For the assumed volume resulting from the spacing of optical CMOS sensors around the experimental object, the result of the trajectory value is always obtained with an accuracy of not less than ±2 [mm] for every frame of the analysis at the frequency 100 [Hz]. In order to eliminate random components in the trajectory signal, a low pass digital filter (5 [Hz]) was used.

The DLT (direct linear transformation) algorithm was employed in order to obtain spatial data by applying a system of four CMOS matrices [24,25]. The *X, Y, Z* coordinates with which the trajectory of the marker attached to the analyzed mechanical object can be obtained, are related to the calculation of the U and V coordinates of the optical sensor. The U and V coordinates are associated with 11 parameters, the values of which are calculated in the calibration procedure:

(1) $$U=\frac{L_{1}X+L_{2}Y+L_{3}Z+L_{4}}{L_{9}X+L_{10}Y+L_{11}Z+1}$$

(2) $$V=\frac{L_{5}X+L_{6}Y+L_{7}Z+L_{8}}{L_{9}X+L_{10}Y+L_{11}Z+1}$$

where: *L*_1_, *L*_11__,_ are optical sensor parameters generated by applying the APAS system.

The *X, Y, Z* coordinate values of any marker attached to the endoscopic tool or the kinematic chain can be specified for one optical sensor as:

(3) $$\left[\begin{array}{ccc} L_{1}-L_{9}U & L_{2}-L_{10}U & L_{3}-L_{11}U\\ L_{5}-L_{9}V & L_{6}-L_{10}V & L_{7}-L_{11}V \end{array}\right]\cdot \left[\begin{array}{c} X\\ Y\\ Z \end{array}\right]=\left[\begin{array}{c} \left(U-L_{4}\right)\\ \left(V-L_{8}\right) \end{array}\right],$$

and for four optical CMOS sensors and given *L*_1,_ …, *L*_11_ parameters used in the experiments as:

(4) $$\left[\begin{array}{ccc} 7.33098-56.35628U^{1} & 1.10437--2.69431U^{1} & -43.38176-1608.7187U^{1}\\ -0.00232-56.35628V^{1} & 0.00001--2.69431V^{1} & -0.00110-1608.7187V^{1}\\ 14.33625-83.80710U^{2} & -1.29458-3.07956U^{2} & 64.38234-1025.0608U^{2}\\ 0.00388-83.80710V^{2} & 0.00003-3.07956V^{2} & 0.00018-1025.0608V^{2}\\ 64.27219-83.95649U^{3} & 64.27219-3.05422U^{3} & 64.27219-1006.6292U^{3}\\ 0.00388-83.95649V^{3} & 0.00003-3.05422V^{3} & 0.00018-1006.6292V^{3}\\ 48.14421-65.05091U^{4} & -3.81080-814.1896U^{4} & 19.95904-2927.1533U^{4}\\ 0.00197-65.05091V^{4} & -0.00026--12.41175V^{4} & -0.00243-2927.1533V^{4} \end{array}\right]\cdot \left[\begin{array}{c} X\\ Y\\ Z \end{array}\right]=\left[\begin{array}{c} \left(U^{1}-6369.3542\right)\\ \left(V^{1}--9.73710\right)\\ \left(U^{2}-582.7693\right)\\ \left(V^{2}-17.06219\right)\\ \left(U^{3}-589.7381\right)\\ \left(V^{3}-17.31318\right)\\ \left(U^{4}-814.1896\right)\\ \left(V^{4}-14.75780\right) \end{array}\right]$$

where: *X, Y, Z* are cartesian coordinates of the marker, *U, V* are coordinates of the optical sensor. Using the matrix (4), it is possible to calculate the position of the CMOS optical sensors in relation to the examined object. The least square method can be applied in computing the coordinates of the markers.

Based on the FDM and trajectories, subsequent physical quantities (i.e., velocity, acceleration and jerk movement) are determinable as approximations of successive time derivatives. The gaps in the trajectory resulting from the loss of visibility of the marker in the CMOS matrix are interpolated using the Lagrange polynomial.

The surgeon’s movements during the in vitro experiment are classified as positioning in relation to the operated heart. The positioning movements were performed by one experienced cardiac surgeon and were aimed at determining the velocities that the surgeon considered as required for obtaining the correct functionality of minimally invasive surgery using endoscopic tools. The correctness of the movement was confirmed by a scientist trained in minimally invasive and robotic techniques. From the point of view of mechanics, the movements should be considered as activities composed of spherical and progressive movements with an imposed constraint in the form of a spherical joint. This means that the surgical robot should be able to move with such velocity values that it can be usefully applied during the operation and be accepted by the surgeon who conducts the operation.

The obtained trajectory waveforms are incorporated as inputs for the created models of the dynamics in surgical robots. To transform the trajectory into joint variables of the robot model, the inverse kinematics problem of position is solved by the method described in article [26]. The discussed method is built upon deriving the transformation matrix between the base of the robot and the effector, and then multiplying both sides of the equation by the inverse matrix that contains the wanted variable—the value of which is important to determine. The inverse kinematics model used is based on obtaining the three vectors of the effector trajectory from the in vitro experiment in relation to the X, Y, and Z axes. Subsequently, the values of the joint variables of the RCM mechanism are deduced.

**Figure 9:**
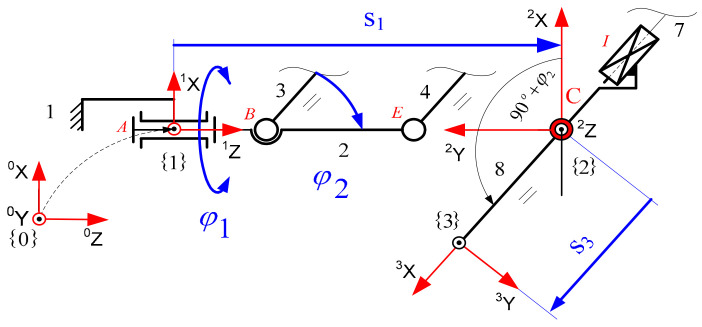
RCM mechanism with marked three joint variables (D-H variables) and a constant point C.

The kinematic chain of a surgical robot with three degrees of freedom is illustrated in Figure 8. The first two degrees of freedom in the robot are responsible for rotational movements $\varphi_{1}$, $\varphi_{2}$. The third degree of freedom is responsible for the translational movement of the endoscopic instrument $s_{3}$. The robot mechanism shown in Figure 9 is called a “remote center of motion (RCM) mechanism”.

**Figure 10:**
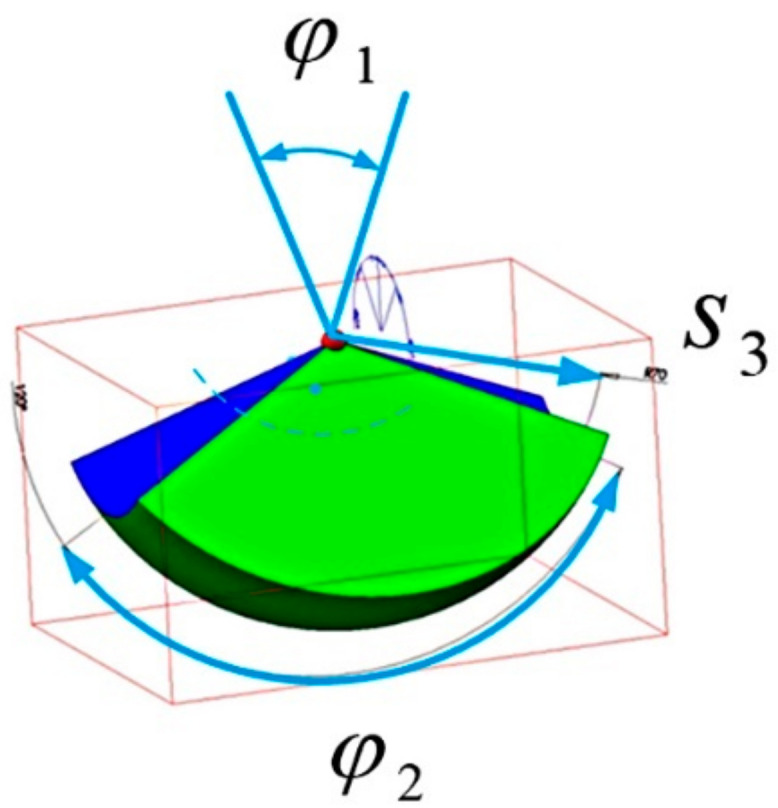
The spherical shape of the working space obtained by the remote center of motion mechanism of a surgical robot. The joints variables are: $\varphi_{1}$, $\varphi_{2}$, $s_{3}$.

The working space obtained by such a mechanism has the shape of a spherical sector. This is illustrated in Figure 10.

Depending on the joint variables of the RCM, the volume of such a workspace (spherical shape) can be strictly defined through an analytical formula as:

(5) $$\left|V\right|=4\overset{\frac{\varphi_{1}}{2}}{\underset{0}{\int }}{d\varphi }_{1}\overset{\frac{\pi }{2}}{\underset{\varphi_{2}}{\int }}\sin(\varphi_{2}){d\varphi }_{2}\overset{\lambda }{\underset{0}{\int }}s_{3}{}^{2}{\mathrm{ds}}_{3}$$

$\varphi_{1}-$ value of the first joint variable of the RCM,

$\varphi_{2}-$ value of the second joint variable of the RCM,

$s_{3}-$ value of the third joint variable of the RCM.

**Figure 11:**
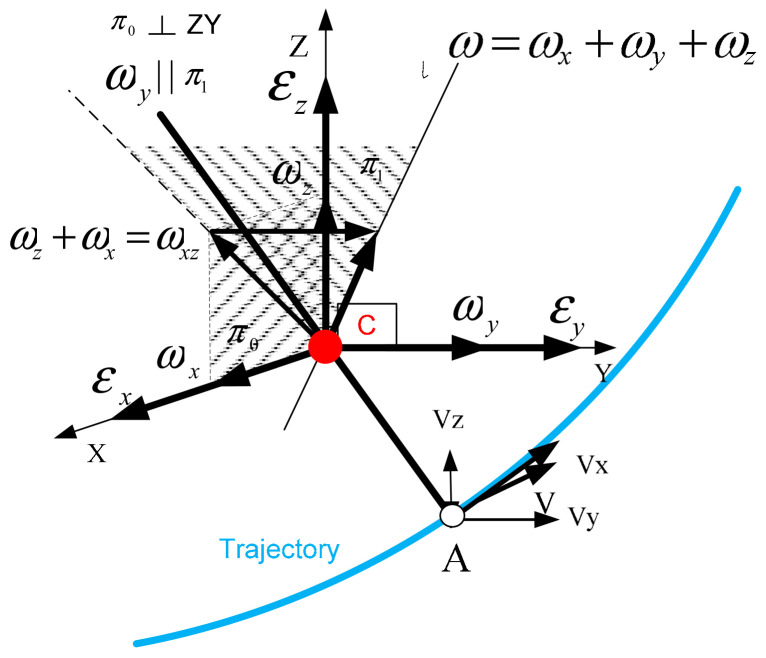
Where: $V_{x}$, $V_{y}$, $V_{z}$ are components of the velocity vector $\vec{V}$ relative to the coordinate system in the constant point C; ω_x_, ω_y_, ω_z_ are components of the rotational velocity vector $\vec{\omega }$ relative to the coordinate system in the constant point C; *r_x_, r_y_, r_z_* are components of radius vector $\vec{r}$ relative to the coordinate system in the constant point C.

On the endoscopic tool used, during the in vitro experiments, restraints are imposed in the form of spherical joints intended to simulate a trocar. The movement of the endoscopic tool after it has passed through the trocar is shown in Figure 11.

The equation for the velocity of point A is defined as:

(6) $$\vec{V}=\vec{\omega }\times \vec{r}=\left[\begin{array}{ccc} 0 & -\omega_{z} & \omega_{y}\\ \omega_{z} & 0 & -\omega_{x}\\ -\omega_{y} & \omega_{x} & 0 \end{array}\right]\left[\begin{array}{c} r_{x}\\ r_{y}\\ r_{z} \end{array}\right]=\left[\begin{array}{c} V_{x}\\ V_{y}\\ V_{z} \end{array}\right],$$

where: $V_{x}$, $V_{y}$, $V_{z}$ are components of the velocity vector $\vec{V}$ relative to the coordinate system in the constant point C; ω_x_, ω_y_, ω_z_ are components of the rotational velocity vector $\vec{\omega }$ relative to the coordinate system in the constant point C; *r_x_*, *r_y_*, *r_z_* are components of radius vector $\vec{r}$ relative to the coordinate system in the constant point C.

The equation for the acceleration of point A is defined as:

(7) $$\vec{a}=\vec{\varepsilon }\times \vec{r}+\vec{\omega }\times \vec{V}=\left[\begin{array}{ccc} 0 & -\varepsilon_{z} & \varepsilon_{y}\\ \varepsilon_{z} & 0 & -\varepsilon_{x}\\ -\varepsilon_{y} & \varepsilon_{x} & 0 \end{array}\right]\left[\begin{array}{c} r_{x}\\ r_{y}\\ r_{z} \end{array}\right]+\left[\begin{array}{ccc} 0 & -\omega_{z} & \omega_{y}\\ \omega_{z} & 0 & -\omega_{x}\\ -\omega_{y} & \omega_{x} & 0 \end{array}\right]\left[\begin{array}{c} V_{x}\\ V_{y}\\ V_{z} \end{array}\right]=\left[\begin{array}{c} a_{x}\\ a_{y}\\ a_{z} \end{array}\right],$$

where: $a_{x}$, $a_{y}$, $a_{z}$ are components of the acceleration vector $\vec{a}$ relative to the coordinate system in the constant point C; *ε_x,_ ε_y,_ ε_z_* are components of the rotational acceleration vector $\vec{\varepsilon }$ relative to the coordinate system in the constant point C.

The movement of the tool after passing through the access port can be described as spherical, which should be superposed with the translational movement reaching any point in the working space shown in Figure 10.

### 4.1. Dynamics Model of a Surgical Robot with RCM Structure Using the Block Diagrams Method

**Figure 12:**
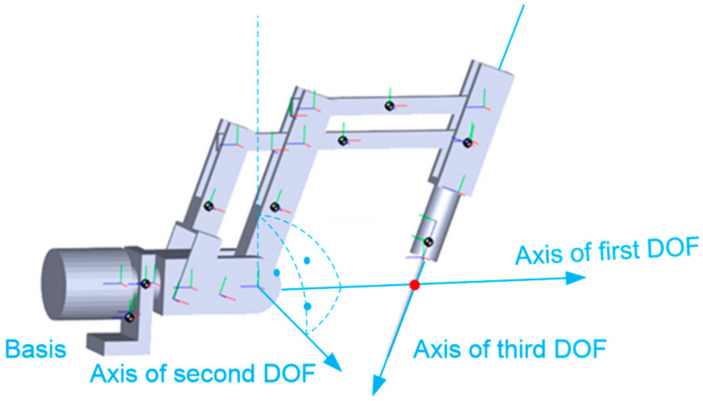
Model of a surgical robot with remote center of motion (RCM) made in the Simulink environment. The joint coordinate systems and the centers of gravity of the links are illustrated. The three axes necessary for obtaining a complex spherical and translational motion are marked. Such movement provides the functionality required during the operation.

The surgical robot dynamics model was built in the Matlab Simulink environment using the block diagram method [27,28] This method was applied to transform the differential equations describing the physical systems of a surgical robot into a block system, where objects are connected by signal transmission lines. An integrating block is used to solving the model. Between the blocks that represent the robot links with specific mass centers and inertia distributions, lines for transmitting the set signal are placed. The set signal values come from the inverse kinematics task of the endoscopic tools positioning. The Simulink environment model of a surgical robot with an RCM with marked joint coordinate systems and centers of gravity that was created in Matlab is shown in Figure 12.

Numerical models of surgical robots contain drive and control systems, therefore, they are highly complex models and quite faithfully correspond to the systems of real objects. Owing to this, the most important criterion for a surgical robot is obtained, which is the safety of its work in the patient’s body.

#### 4.1.1. Model of DC Motor

DC motors are often employed as drives for surgical robots. They are used, for example, in the American construction of “da Vinci”—currently, the most widely employed clinical surgical robot. The analytical equations of the DC motor for a continuous time have the following form:

(8) $$\frac{d}{dt}\cdot \left[\begin{array}{c} \frac{d\omega }{dt}\\ i \end{array}\right]=\left[\begin{array}{cc} -\frac{b}{J} & \frac{k}{J}\\ -\frac{k}{L} & -\frac{R}{L} \end{array}\right]\cdot \left[\begin{array}{c} \omega \\ i \end{array}\right]+\left[\begin{array}{c} 0\\ \frac{1}{L} \end{array}\right]\cdot U$$

(9) $$y=\left[\begin{array}{cc} 1 & 0 \end{array}\right]\cdot \left[\begin{array}{c} \omega \\ i \end{array}\right]$$

wherein:

*k*—back electromotorical force coefficient,

*i*—current,

*L*—inductance,

ω—rotational velocity,

*R*—resistance,

*b*—viscous coefficient,

*U*—voltage.

**Figure 13:**
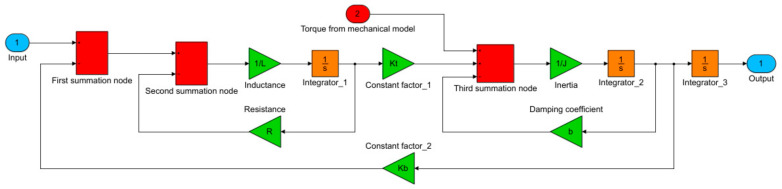
Model of a DC motor made by the method of block diagrams. A signal was added to the motor model with the value of the driving torque needed to realize the dynamics of the remote center of motion mechanism of the surgical robot.

The block diagram, which was created based on Equations (8) and (9) describing the DC motor model is illustrated in Figure 13. Each surgical robot joint is driven by this actuator.

#### 4.1.2. PID Controller Model

A PID controller was added to the system with a feedback in each joint. The PID controller model is described by the equation:

(10) $$u\left(t\right)=k_{p}\left(\varepsilon \left(t\right)+\frac{1}{T_{i}}\overset{t}{\underset{0}{\int }}\varepsilon \left(\tau \right)d\tau +T_{d}\frac{d\varepsilon \left(t\right)}{dt}\right)$$

wherein:

$u\left(t\right)$—control,

$k_{p}$—proportional gain,

$T_{i}$—integration time,

$T_{d}\;$—derivative time,

$\varepsilon \left(t\right)\;$—error time.

**Figure 14:**
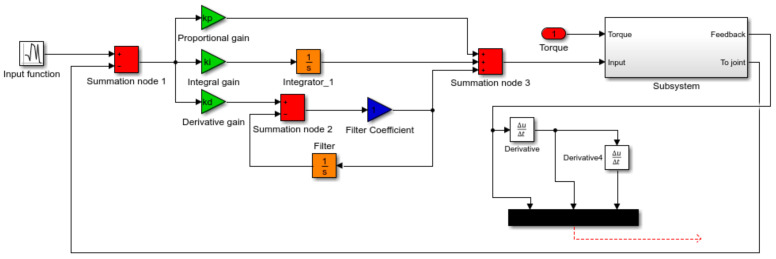
The PID controller model created by the block diagram method. The dashed line illustrates the connection to the joint of the surgical robot model.

The block diagram, which was created on the basis of Equation (10), describing the PID controller model is illustrated in Figure 14.

The model of surgical robot created in Matlab/Simulink is solved using the ODE45 (ordinary differential equations) function by application of the Dormand–Prince method.

### 4.2. Dynamical Model of Transient States Using FEM

**Figure 15:**
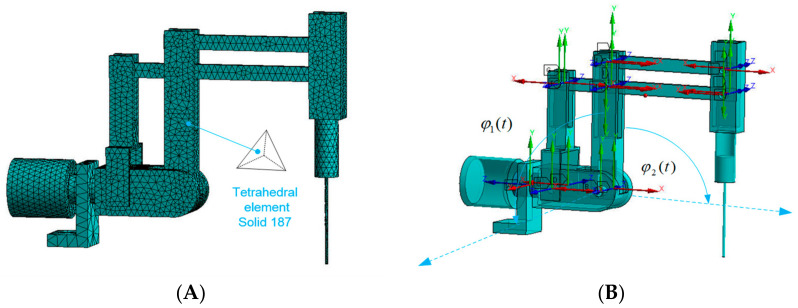
(**A**) Mesh model of the surgical robot. Discretization was done through applying the Solid 187 tetrahedral element. (**B**) Model with motion in transient states.

The transient dynamics model was built using the FEM mesh method. This mesh model has 11,832 degrees of freedom. The discretization is performed through applying the Solid 187 element [29]. The motion model of the robot was created from links connected by kinematic rotational pairs. Both structures are shown in Figure 15.

The FEM model makes it possible to obtain motion strength values. Deformations resulting from the time of the previous analysis and influencing the later moment as well as inertial interactions are taken into account. Inputs from the experiment on a cardiovascular tissue with the maximum value for the positioning movement in relation to the heart tissue are also applied to the model. Movement is carried out in the first degree of freedom around an axis parallel to the ground. Movement is carried out in the second degree of freedom in such a method that the endoscope during the entire numerical analysis is in the plane perpendicular to the ground. The data can be applied to numerical models of the dynamics of driving moments and deformations.

## 5. Results

**Figure 16:**
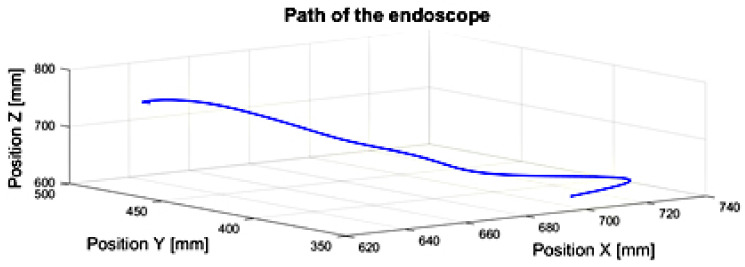
The path of the endoscopic tool during the positioning movement after passing through a trocar simulated with a spherical joint in relation to the heart tissue. The tool has four degrees of freedom.

Based on the trajectory of the endoscopic tool during the tool positioning movement against the heart tissue in the in vitro surgery, other kinematic parameters of the tools, important from the point of view of endoscopic surgery, were determined. The trajectory of the endoscopic tool is illustrated in Figure 16. The tool passes through a simulated spherical trocar joint. The surgeon does not directly observe the heart tissue, rather, the image of the heart is delivered to the monitor by the endoscopic camera of the surgical robot, “Robin Heart Vision”. Based on the obtained results, it is possible to objectively assess the surgeon’s technical abilities to perform endoscopic surgery.

**Figure 17:**
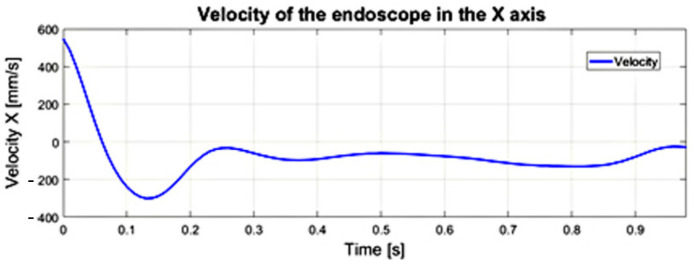
Velocity course of the endoscopic tool along the X−axes obtained on the basis of the trajectory.

The course of velocity resulting from the application of the FDM to the measurement data of the trajectory of endoscopic tools is illustrated in Figure 17. The velocity values of the endoscopic instrument are easy to interpret as they do not require the surgeon to recognize any mathematical apparatus. Smooth slopes are observed on the course of the velocity, which proves that during the tool positioning movements with the operated tissue, there are no sudden changes in velocity resulting from the tool positioning requirements during the operation. The maximum amplitude of the obtained speed course was about 600 [mm/s].

**Figure 18:**
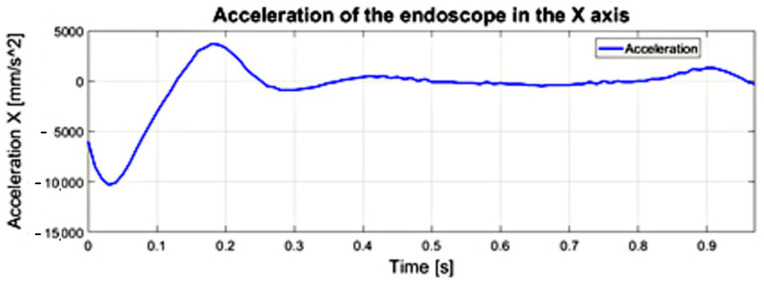
The acceleration course of the endoscopic tool along the *X*−axis. The figure illustrates the acceleration fluctuations.

From 0.4 [s] onwards on the acceleration curve, which allows inferring the dynamics of the endoscopic tool, acceleration fluctuations can be observed. The fluctuations in acceleration shown in Figure 18 are related to the mobility (vibrations) of the trocar simulated with a spherical joint and vibrations of the surgeon’s hand−trocar−endoscopic instrument system. In order to minimize this phenomenon, it would be necessary to stiffen the system simulating the trocar or to stiffen the attachment of the trocar to the human body. The surgeon’s hand, which is subject to tremor, can be replaced with a surgical robot, which reduces the tremor and vibrations of the endoscopic tool−trocar−tissue system due to the structural systems that stiffen the cooperating systems.

**Figure 19:**
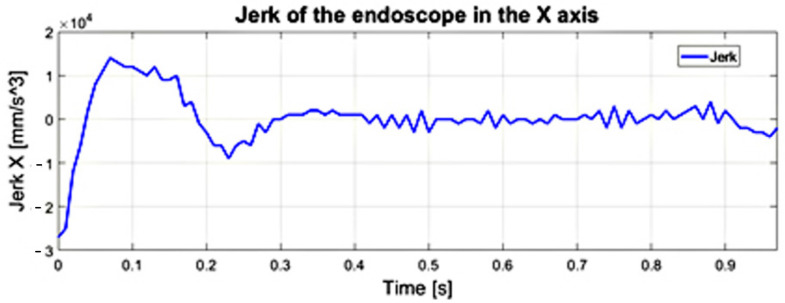
The jerk-like course of the endoscopic tool along the *X*−axis obtained on the basis of the course of acceleration—which is not constant in terms of value.

The course of the jerk-type movement, understood as the third time derivative of the trajectory, from which it is possible to reveal involuntary swift, sharp, sudden movements of the endoscopic tool that may occur while positioning the endoscopic tool in relation to the heart tissue—is illustrated in Figure 19. The appearance of a spurt in the structure of the endoscopic instrument—trocar-surgeon’s hand proves the lack of fluidity of the movement performed by the surgeon.

**Figure 20:**
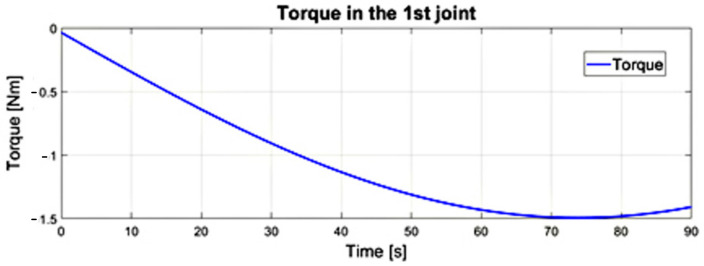
Waveform of the driving torque in the first joint from the dynamics model of the surgical robot in Simulink.

**Figure 21:**
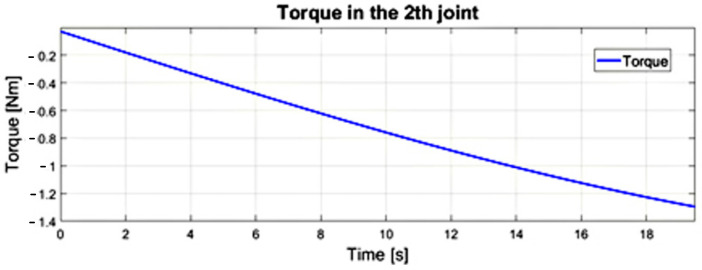
Waveform of the driving torque in the second joint from the dynamics model of the surgical robot in the Simulink.

The value of the driving torque in the first degree of freedom of the numerical model made in the Simulink program for the input equals to ω_1_ = 1.3 [deg/s]. This is determined as the maximum during the positioning movement in relation to the heart tissue after the endoscope passes through the access port, which is illustrated in Figure 20. Herein, the maximum value of ω_2_ was 2.75 [deg/s], while $v_{3}$ was equal to 0.27 [cm/s]. The course of the driving torque in the second degree of freedom is illustrated in Figure 21. The paths of the driving torque in the first and second degrees of freedom are periodic. Based on the obtained driving torque, it is possible to select the driving system of a surgical robot with a remote center of motion mechanism.

For positioning activities, when the tool is outside the body cavity or unambiguously pulled out from the body, the velocities are: ω_1_ = 14.78 [deg/s], ω_2_ = 35.96 [deg/s], $v_{3}$ = 9.43 [deg/s].

**Figure 22:**
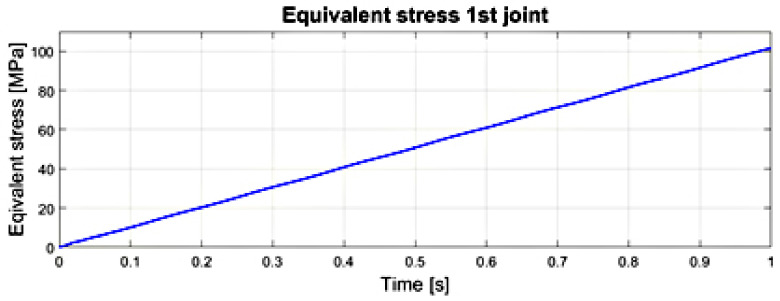
The course of stress reduced according to the von Mises hypothesis during the analysis of transient states—movement in the first joint of surgical robot.

**Figure 23:**
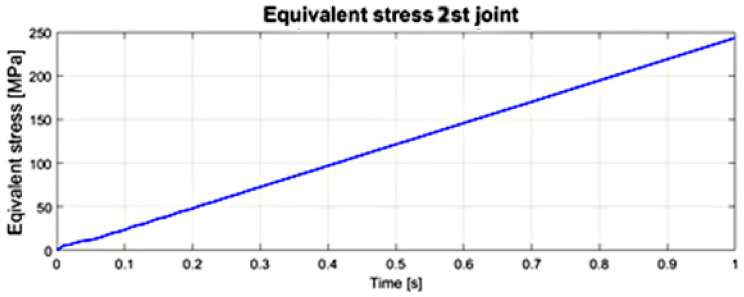
The course of stress reduced according to the von Mises hypothesis during the analysis of transient states—movement in the second joint of surgical robot.

The courses of the maximum values of stress reduced according to the von Mises hypothesis, obtained at any time in the transient analysis of the numerical model in the first and second joints of the surgical robot are illustrated in Figure 22 and Figure 23. The significance of the reduced stress is substantial in the case of the surgical robot’s effort (danger of reaching a critical state) by reaching the yield point in the case of a complex stress state. The von Mises hypothesis, where the effort is measured by the shear strain energy, gives results close to the experimental and is applied to elastic-plastic materials [30,31].

Reduced stress *σ*_red._ (equivalent) is the stress in the uniaxial stretching state, at which the magnitude of the shear strain energy reaches the same value as in a specific complex stress.

The transient motion analysis is tested at the rotational velocity of 1.3 [rad/s] in the first joint. Throughout the analysis, the robot endoscope moves around an axis parallel to the ground. The movement in the second degree of freedom takes place in the plane perpendicular to the ground at the speed of 2.75 [rad/s].

**Figure 24:**
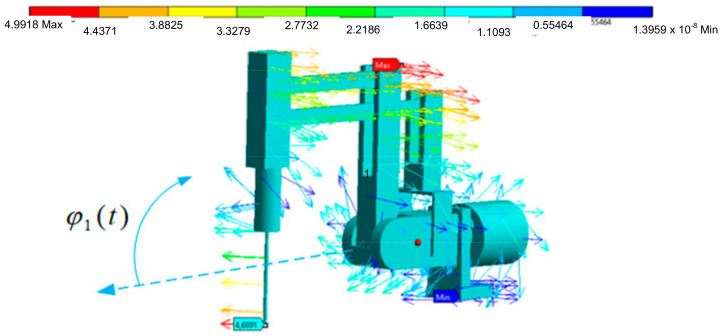
Displacement vector diagram in [mm] for the movement of the surgical robot at the first joint.

**Figure 25:**
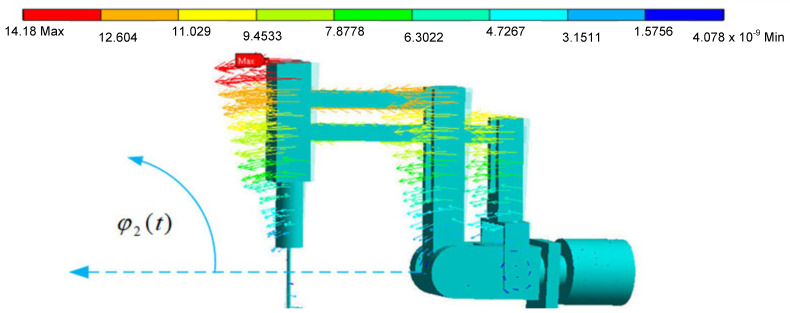
Displacement vector diagram in [mm] for the movement of the surgical robot at the second joint.

The endoscope displacement vector charts have maximum values for the maximum distances from the axis of rotation, which is a feature of spherical kinematics of remote center of motion mechanisms. These values are illustrated in Figure 24 and Figure 25. The displacement is given in [mm].

Based on Figure 24 and Figure 25, it is possible to evaluate the resultant displacement of the endoscope during transient states resulting from the displacement and deformation of the surgical robot structure. The obtained course of reduced stress enables the determination of the safety factor value in the surgical robot structure (the safety of the surgical robot from the strength point of view). Such an obtained model makes it possible to repeatedly test the value of the safety factor after changing the cross-sections of the robot’s profiles without the need for long-term and costly experimental tests.

## 6. Discussion

In this study, it was possible to achieve the values of the waveforms of kinematic quantities using four CMOS optical sensors and the DLT algorithm. These served as data for models of the surgical robot dynamics in driving moments and the dynamics of transients. By means of the obtained data, it is also possible to observe what strength values (stress, displacement) appear in the structure of a surgical robot during in vitro operations on heart tissue.

It is necessary to experimentally obtain input signals to numerical models of dynamics in surgical robots to receive interactions close to those that actually appear in the structures. From the utilitarian point of view, it is worth analyzing qualitatively the courses of physical quantities that appear in the structure of a surgical robot as a result of the actual loads resulting from the surgeon’s movements in order to predict the safety margin (for example, from the point of view of the long-term fatigue of the surgical robot structure), resulting from the applicable national safety standard.

The significant disadvantage of the method with the use of optical sensors includes the limited volume of the experiment. As the volume of the experiment increases, the accuracy of the obtained trajectory decreases. The second important limitation of the optical method is the inability to record fast-changing signals and signals with relatively small amplitudes. It is best to determine the experimental test for a given arrangement of optical CMOS sensors in the volume of the experiment for which the accuracy of the obtained result was previously determined. The disadvantage of using the optical system is the loss of the trajectory in the case of the disappearance of the visibility of the marker in the lens of the optical sensor. The gap in the trajectory can, however, be interpolated by applying the Lagrange polynomial, which was done in this paper.

The currently used dynamics models are equipped with elements of drive systems, regulators, and loads related to resistance in motion, hence adding inputs consistent with reality in a very realistic way. This produces outcomes in the form of the waveforms of the various mechanical quantities. The values of the driving torque that will be generated by the driving servomotors can also be noted. Such a procedure clearly allows the size of the drive systems to be minimized, therefore it is justified from the perspective of surgical robot design.

The block diagram method in the Simulink program makes it possible to apply the approach to any mechanical system. Furthermore, drive systems and control systems can be easily added to the mechanical structure. The approach revealed in this paper is an alternative method to one based upon matrix transformations and numerical solution of differential equations. The authors also express the view that the use of the Simulink program significantly shortens the time of obtaining a numerical model with significant complications, in relation to methods employing matrix transformations and difference equations.

## 7. Conclusions

Based on the obtained results, it is possible to objectively assess a surgeon’s technical abilities to perform endoscopic surgery. At the same time, data can be applied to numerical models of the dynamics of driving moments and deformations.

Research with the use of four optical CMOS Basler A602fc-2 sensors was carried out on a real object biokinematic chain involving Robin Heart, Robin Heart Vision, endoscopic tools, and a surgeon, through an experiment utilizing cardiovascular tissue. Due to the application of commercial APAS software and the DLT algorithm, it was possible to obtain the trajectories of endoscopic instruments that are useful for generating dynamic models.

In the work, waveforms of displacement time derivatives were generated based on the data from sensors after applying appropriate filtering from random components and following the application of FDM. We demonstrated that the obtained values of kinematic waveforms of endoscopic tools are suitable as inputs for the created models of dynamics of a surgical robot. The courses of trajectories, velocities, accelerations, jerk-movement, torques, reduced stresses and displacements for endoscopic tools were especially obtained. The shape of the resulting trajectories allows for evaluating the complication of the cardiac surgery performed. On the basis of the course of the jerk-movement, there was a lack of smoothness in the actions of the cardiac surgeon controlling the endoscopic instruments after their passage through trocars simulated with spherical joints.

It is worth emphasizing that the obtained data became the input data for two models of dynamics of surgical robots. The first model concerns the dynamics of the driving moments in a surgical robot and is built in the Simulink environment. It enables selecting drive systems based on a DC motor in a system controlled by a PID regulator. The second model is based on the finite element method and von Mises hypothesis, and enables gaining strength inference during the numerical analysis of transient states. By means of the inputs from the in vitro experiment, it is possible to obtain reliable measurements of various physical quantities (stress, displacement, torque, safety factor) that appear in the structure of a surgical robot as generated through applying either model.

Subsequent work will be aimed at conducting experiments using CMOS sensors for determining kinematic values of endoscopic tools in the subject of abdominal cavity, urology, and neurology.

## Data Availability

Not applicable.
